# Special Issue on Enterotoxigenic *Escherichia coli* (ETEC) Vaccines: ETEC Infection and Vaccine-Mediated Immunity

**DOI:** 10.3390/microorganisms12061087

**Published:** 2024-05-27

**Authors:** Frederick J Cassels, Ibrahim Khalil, A. Louis Bourgeois, Richard I Walker

**Affiliations:** 1 PATH, Seattle, WA 98109, USA; 2 Department of Global Health, University of Washington, Seattle, WA 98105, USA; ikhalil@uw.edu; 3 PATH, Washington, DC 20001, USA; lbourge1@jhu.edu (A.L.B.); rwalker2019@gmail.com (R.I.W.)

## 1. Introduction

Enterotoxigenic *Escherichia coli* (ETEC) is the most prevalent bacterial pathogen causing young children to suffer acute watery diarrhea in Low- and Middle-Income Countries (LMICs). This high ETEC burden results in children facing numerous diarrhea episodes, contributing to poor growth and cognitive development. ETEC also remains the leading cause of diarrhea among travelers and military personnel visiting endemic areas. The WHO recently reaffirmed that ETEC is a vaccine priority, and, given its recognition as a significant Anti-Microbial Resistance (AMR) threat, public health stakeholders have urged the acceleration of the development of a vaccine [1,2].

ETEC is often the first enteric illness encountered by many infants; thus, it is important to induce protective immunity by the age of 9 months to cover peak incidence and mortality at least throughout the first 24 months of life. In addition to potential direct, individual effects on ETEC mortality and morbidity, an ETEC vaccine is also likely to have significant indirect effects, such as decreasing antibiotic use and thereby reducing the prevalence of AMR bacteria; increasing herd protection at the community level; increasing healthcare cost savings by preventing malnutrition; and improving child physical and cognitive development. Protection from all-cause diarrhea may also be observed, a phenomenon that has been seen with use of rotavirus vaccines. Although several ETEC vaccine candidates have been tested and are in different stages of product development, there are no licensed vaccines against ETEC-induced diarrhea [1,2].

The aim of this Special Issue of *Microorganisms* is to present articles that provide an in-depth look at the current state of ETEC vaccines and the supportive information required to ultimately advance these vaccines to licensure. The topics solicited for this Special Issue include epidemiology and global burden; host parameters and genomics that predict responses; the application of new omics technologies for the characterization of host responses; the preclinical evaluation of vaccine candidates and models of disease; vaccine candidates in clinical trials and human challenge models; and the assessment of the value of vaccines. The sixteen contributions are listed in Table 1 below. It is essential to obtain a broad distribution of study sites and geographic regions from which samples are derived, with twelve countries represented in this case, as ETEC is a global disease.

1. Héma, A.; Sermé, S.S.; Sawadogo, J.; Diarra, A.; Barry, A.; Ouédraogo, A.Z.; Nébié, I.; Tiono, A.B.; Houard, S.; Chakraborty, S.; et al. Contribution of the Rapid LAMP-Based Diagnostic Test (RLDT) to the Evaluation of Enterotoxigenic *Escherichia coli* (ETEC) and *Shigella* in Childhood Diarrhea in the Peri-Urban Area of Ouagadougou, Burkina Faso. *Microorganisms* **2023**, *11*, 2809. https://doi.org/10.3390/microorganisms11112809 (accessed on 10 March 2024).
2. Mwape, K.; Bosomprah, S.; Chibesa, K.; Silwamba, S.; Luchen, C.C.; Sukwa, N.; Mubanga, C.; Phiri, B.; Chibuye, M.; Liswaniso, F.; et al. Prevalence of Diarrhoeagenic *Escherichia coli* among Children Aged between 0–36 Months in Peri-Urban Areas of Lusaka. *Microorganisms* **2023**, *11*, 2790. https://doi.org/10.3390/microorganisms11112790 (accessed on 10 March 2024).
3. Chakraborty, S.; Johura, F.-T.; Sultana, M.; Zhang, X.; Sadique, A.; George, C.M.; Monira, S.; Sack, D.A.; Sack, R.B.; Alam, M. Epidemiology of Enterotoxigenic *Escherichia coli* among Children and Adults Seeking Care at Hospitals in Two Geographically Distinct Rural Areas in Bangladesh. *Microorganisms* **2024**, *12*, 359. https://doi.org/10.3390/microorganisms12020359 (accessed on 10 March 2024).
4. Johura, F.-T.; Sultana, M.; Sadique, A.; Monira, S.; Sack, D.A.; Sack, R.B.; Alam, M.; Chakraborty, S. The Antimicrobial Resistance of Enterotoxigenic *Escherichia coli* from Diarrheal Patients and the Environment in Two Geographically Distinct Rural Areas in Bangladesh over the Years. *Microorganisms* **2024**, 12, 301. https://doi.org/10.3390/microorganisms12020301 (accessed on 10 March 2024).
5. Kuhlmann, F.M.; Grigura, V.; Vickers, T.J.; Prouty, M.G.; Iannotti, L.L.; Dulience, S.J.L.; Fleckenstein, J.M. Seroprevalence Study of Conserved Enterotoxigenic *Escherichia coli* Antigens in Globally Diverse Populations. *Microorganisms* **2023**, *11*, 2221. https://doi.org/10.3390/microorganisms11092221 (accessed on 10 March 2024).
6. Girardi, P.; Bhuiyan, T.R.; Lundin, S.B.; Harutyunyan, S.; Neuhauser, I.; Khanam, F.; Nagy, G.; Szijártó, V.; Henics, T.; Nagy, E.; et al. Anti-Toxin Responses to Natural Enterotoxigenic *Escherichia coli* (ETEC) Infection in Adults and Children in Bangladesh. *Microorganisms* **2023**, *11*, 2524. https://doi.org/10.3390/microorganisms11102524 (accessed on 10 March 2024).
7. Hollifield, I.E.; Motyka, N.I.; Fernando, K.A.; Bitoun, J.P. Heat-Labile Enterotoxin Decreases Macrophage Phagocytosis of Enterotoxigenic *Escherichia coli*. *Microorganisms* **2023**, *11*, 2121. https://doi.org/10.3390/microorganisms11082121 (accessed on 10 March 2024).
8. Brønstad, I.; von Volkmann, H.L.; Sakkestad, S.T.; Steinsland, H.; Hanevik, K. Reduced Plasma Guanylin Levels Following Enterotoxigenic *Escherichia coli*-Induced Diarrhea. *Microorganisms* **2023**, *11*, 1997. https://doi.org/10.3390/microorganisms11081997 (accessed on 10 March 2024).
9. Mwape, K.; Mubanga, C.; Chilyabanyama, O.N.; Chibesa, K.; Chisenga, C.C.; Silwamba, S.; Randall, A.; Liang, X.; Barnard, T.G.; Simuyandi, M.; et al. Application of a Novel Proteomic Microarray Reveals High Exposure to Diarrhoeagenic *Escherichia coli* among Children in Zambia Participating in a Phase I Clinical Trial. *Microorganisms* **2024**, *12*, 420. https://doi.org/10.3390/microorganisms12030420 (accessed on 10 March 2024).
10. Li, S.; Seo, H.; Upadhyay, I.; Zhang, W. A Polyvalent Adhesin–Toxoid Multiepitope-Fusion-Antigen-Induced Functional Antibodies against Five Enterotoxigenic *Escherichia coli* Adhesins (CS7, CS12, CS14, CS17, and CS21) but Not Enterotoxins (LT and STa). *Microorganisms* **2023**, *11*, 2473. https://doi.org/10.3390/microorganisms11102473 (accessed on 10 March 2024).
11. Westcott, M.M.; Blevins, M.; Wierzba, T.F.; Morse, A.E.; White, K.R.; Sanders, L.A.; Sanders, J.W. The Immunogenicity and Properties of a Whole-Cell ETEC Vaccine Inactivated with Psoralen and UVA Light in Comparison to Formalin. *Microorganisms* **2023**, *11*, 2040. https://doi.org/10.3390/microorganisms11082040 (accessed on 10 March 2024).
12. Maier, N.; Grahek, S.L.; Halpern, J.; Restrepo, S.; Troncoso, F.; Shimko, J.; Torres, O.; Belkind-Gerson, J.; Sack, D.A.; Svennerholm, A.-M.; et al. Efficacy of an Enterotoxigenic *Escherichia coli* (ETEC) Vaccine on the Incidence and Severity of Traveler’s Diarrhea (TD): Evaluation of Alternative Endpoints and a TD Severity Score. *Microorganisms* **2023**, *11*, 2414. https://doi.org/10.3390/microorganisms11102414 (accessed on 10 March 2024).
13. Talaat, K.R.; Porter, C.K., Chakraborty, S., Feijoo, B., Brubaker, J., Ajoodani, B.A., DeNearing, B., Prouty, M.; Poole, S., Bourgeois, A.L., Billingsley, M., Sack, D., Lingelbach, S.E., Taucher, C. Validation of a human challenge model using an LT-expressing Enterotoxigenic *E. coli* strain (LS03-016011) and characterization of potential amelioration of disease by an investigational oral LT-ETEC vaccine candidate (VLA1701). *Microorganisms* **2024**, https://doi.org/10.3390/microorganisms12040727 (accessed on 10 March 2024).
14. Gutiérrez, R.L.; Riddle, M.S.; Porter, C.K.; Maciel, M., Jr.; Poole, S.T.; Laird, R.M.; Lane, M.; Turiansky, G.W.; Jarell, A.; Savarino, S.J. A First in Human Clinical Trial Assessing the Safety and Immunogenicity of Two Intradermally Delivered Enterotoxigenic *Escherichia coli* CFA/I Fimbrial Tip Adhesin Antigens with and without Heat-Labile Enterotoxin with Mutation LT(R192G). *Microorganisms* **2023**, *11*, 2689. https://doi.org/10.3390/microorganisms11112689 (accessed on 10 March 2024).
15. Gutiérrez, R.L.; Porter, C.K.; Harro, C.; Talaat, K.; Riddle, M.S.; DeNearing, B.; Brubaker, J.; Maciel, M., Jr.; Laird, R.M.; Poole, S.; et al. Efficacy Evaluation of an Intradermally Delivered Enterotoxigenic *Escherichia coli* CF Antigen I Fimbrial Tip Adhesin Vaccine Coadministered with Heat-Labile Enterotoxin with LT(R192G) against Experimental Challenge with Enterotoxigenic *E. coli* H10407 in Healthy Adult Volunteers. *Microorganisms* **2024**, *12*, 288. https://doi.org/10.3390/microorganisms12020288 (accessed on 10 March 2024).
16. Hossain, M.J.; Svennerholm, A.-M.; Carlin, N.; D’Alessandro, U.; Wierzba, T.F. A Perspective on the Strategy for Advancing ETVAX^®^, An Anti-ETEC Diarrheal Disease Vaccine, into a Field Efficacy Trial in Gambian Children: Rationale, Challenges, Lessons Learned, and Future Directions. *Microorganisms* **2024**, *12*, 90. https://doi.org/10.3390/microorganisms12010090 (accessed on 10 March 2024).

## 2. Epidemiology and Global Burden

A deeper understanding of the global burden of a pathogen is critical for allocating limited resources to address public health priorities. Contribution 1 describes the application of the rapid Loop-Mediated Isothermal Amplification (LAMP) diagnostic test (RLDT) to stool samples from children under five years of age from Burkina Faso, West Africa. RLDT demonstrated high sensitivity and specificity compared to cultur followed by PCR. Of the 165 stool samples analyzed for ETEC, 30% were positive when using RLDT, while 4.2% were positive (constituting a 5.9-fold difference) when using cultur followed by PCR. These results likely indicate substantial underdiagnosis and therefore a potential to improve the accuracy of ETEC disease burden estimation, particularly for this region of Africa.

Using samples from five peri-urban health facilities in Lusaka, Zambia, Contribution 2 examined stool samples from 0–3-year-old children old presenting with diarrhea. Of the 590 samples tested, 76% contained diarrheagenic *E. coli*, with 30% containing ETEC, 40% containing enteroaggregative *E. coli*, and 45% containing enteropathogenic *E. coli*. In total, 50% of the diarrheagenic *E. coli*-positive samples comprised multiple pathotypes of varying virulence gene combinations. High prevalence of diarrheagenic *E. coli* is observed in the early exposure (<12 months) of children to enteric *E. coli*.

In Contribution 3, the authors searched for ETEC in diarrhea samples from children and adults seeking hospital care in two distinct areas in rural Bangladesh, specifically Chhatak in the north and Mathbaria in the southern coastal area. ETEC was highly prevalent in both areas, though the proportions of toxin types and colonization factors (CF) varied by location, season, and age group. Children under five years of age and adults 20–60 years of age had the highest risk of being afflicted with ETEC-induced diarrhea requiring urgent care.

Working in the same Bangladeshi geographical locations as Contribution 3, the authors of Contribution 4 tested ETEC from diarrheal and rectal swab samples and collected samples from rivers, ponds, and canals to analyze antimicrobial resistance profiles. Multidrug-resistant (MDR) ETEC strains were found in 60% of the Mathbaria and 72% of the Chhatak diarrhea samples, and they were also found in 69% and 30% of environmental samples from Mathbaria and Chhatak, respectively.

## 3. Host Parameters and Genomics That Predict Responses

Gaining a deeper understanding of the immune responses elicited by ETEC vaccine candidates and induced by natural infections, including the wide array of antigens the immune response targets, is key to designing and implementing a successful vaccine. The authors of Contribution 5 screened serum samples from Egypt, Cameroon, Peru, Haiti, and the US for IgG and IgA responses to LT and the non-canonical ETEC components EatA and EtpA. The results revealed elevated levels of both antibody isotypes in subjects from endemic regions for all three antigens, supporting further evaluation of EatA and EtpA as candidate ETEC vaccine components.

In Contribution 6, the authors screened diarrhea patient sera from adults and children from Dhaka, Bangladesh, for anti-toxin responses. Fewer than 25% of the subjects generated an anti-LT response, which was neutralizing for both LT and Cholera toxin (CT). Furthermore, it was determined that the antibody reactive to LT linear epitopes was non-neutralizing.

Contribution 7 reported on the impact of LT and ST enterotoxins on the phagocytosis of ETEC by cultured murine macrophages. They demonstrated that LT but not ST intoxication decreased the number of ETEC phagocytized by macrophages. Macrophages exposed simultaneously to LPS and LT produced IL-33, a cytokine implicated in promoting macrophage alternative activation, iron recycling, and intestinal repair. These data demonstrate that LT provides ETEC with the ability to decrease the perceived ETEC burden and suppresses the initiation of inflammation, suggesting that host IL-33/IL-33R signaling may augment pathways that promote iron restriction to facilitate ETEC escape from macrophages. These results could explain novel mechanisms of immune subversion that may ultimately contribute to asymptomatic ETEC carriage.

Reduced plasma guanylin (GN) levels following ETEC diarrhea were present in subjects evaluated in a Controlled Human Infection Model (CHIM) study, as demonstrated by Contribution 8. Twenty-one volunteers were experimentally infected with ETEC, with blood and urine specimens obtained at 0 to 7 days after the challenge. Ten subjects developed diarrhea (D), and eleven did not (ND). The D group’s plasma pro GN, and not prouroganylin (UGN), concentrations were substantially reduced two to three days after the onset of diarrhea, with no changes seen in the ND group. ETEC diarrhea seemed to affect diauresis, the zinc/creatine ratio, and sodium and chloride secretion in the urine. Reductions in plasma pro GN levels could serve as a marker for intestinal isotonic fluid loss.

## 4. Application of New Omics Technologies for the Characterization of Host Responses

Contribution 9 tested a new pan-diarrheagenic *E. coli* proteomic array consisting of 2168 proteins from enterohemmorhagic (EHEC), enteroinvasive (EIEC), enteropathogenic (EPEC), and extraintestional *E. coli* (ExPEC), all of which complement the previously tested ETEC proteomic array. Serum samples from a Zambian phase 1 ETVAX (Table 2) clinical trial were tested. The results indicated that all the subjects had high levels of exposure to Ipa, SseC, and EspB proteins, suggesting high levels of exposure to EPEC, EHEC, EAEC, EIEC, and ETEC in this Zambian population. These results are consistent with the results of recent epidemiological studies assessing the etiology of diarrheal disease in infants and young children in Zambia.

## 5. Preclinical Evaluation of Vaccine Candidates and Models of Disease

Contribution 10 generated a polyvalent adhesin-toxoid multiepitope-fusion-antigen (MEFA IIb) containing epitopes of five ETEC adhesins, namely, CS7, CS12, CS14, CS17, and CS21, and of LT and Sta. MEFA-IIb induced the production of antibodies in mice that inhibited adherence from all ETEC adhesins and reduced enterotoxigenicity of STa. Immunogenicity and functional antibody inhibited the adherence of ETEC strains expressing any of these five adhesins but failed to neutralize ST or LT enterotoxicity. IM-Immunized rabbits developed robust antigen-specific antibodies when challenged with an ETEC isolate expressing CS21 and STa, presenting a significant reduction in intestinal colonization by ETEC bacteria. The results thus indicated that MEFA IIb was broadly immunogenic against adhesins but did not generate a functional response against toxins.

A novel whole-cell inactivation vaccine technology was developed by the authors of Contribution 11 and applied to ETEC whole cells while retaining the antigenicity of proteins throughout the process. Psoralen and UVA light (PUVA), which target bacterial nucleic acid, treatment yielded the replication-incompetent ETEC strain H10407. The anti-ETEC IgG titers generated were similar for the PUVA and comparator-formalin-treated vaccines; however, the levels of IgG against several conserved ETEC antigens were greater after vaccination with PUVA-ETEC.

## 6. Vaccine Candidates in Clinical Trials and Human Challenge Models

The recently developed disease severity score and alternative clinical endpoints were evaluated in Contribution 12 as part of a validation effort undertaken to assess the efficacy of the earlier formulation of ETVAX in first-time travelers to an ETEC-endemic area. Data on 1434 travelers to Guatemala or Mexico who had been vaccinated with the ETVAX ETEC vaccine indicated that subjects with serum IgA titers corresponding to CTB had a lower risk of infection with ETEC and Campylobacter. In addition, it was reported that the Travelers’ diarrhea (TD) severity score provided a more robust descriptor of disease severity and should be included as an endpoint in future studies.

Contribution 13 reported on work conducted to validate the human challenge model LSN03, an LT-only-expressing ETEC strain. A challenge with this strain induced moderate-to-severe diarrhea in 73% of recipients. Vaccine recipients given the Valneva ETEC vaccine (Table 2) tended to shed lower levels of challenge strain compared to non-recipients (*p* = 0.056), and their disease severity scores were lower.

Two intradermally (ID) delivered ETEC CFA/I fimbrial tip adhesin antigens (FTA) and the LT (R192G) single-mutant mucosal adjuvant (mLT) were administered (three doses in three-week intervals) in a first- in-human phase 1 clinical trial (Table 2) reported by Contribution 14. Serum and antibody-secreting cell (ASC) responses were measured. High serologic and ASC responses were seen across the study groups, with the most robust vaccinated with 25 ug of the dscCfaE fimbrial tip antigen with 0.1 ug of mLT. Both dscCfaE constructs with mLT were found to be safe and immunogenic.

Contribution 15 evaluated the efficacy of an ID-delivered ETEC CFA/I FTA vaccine with mLT (Table 2), for which the optimal dose was determined in Contribution 14, against an experimental challenge with ETEC strain H10407 in healthy adults. The subjects were assessed for moderate-to-severe diarrhea (MSD) for 5 days post challenge, while the H10407 attack rate was 45.5 to 64.7% across cohorts. The vaccine recipients had lower loose stool output and MSD, with an overall vaccine protective efficacy of 27.8%. This efficacy improved against more severe forms of ETEC illness, as determined via the application of a disease severity score.

In a perspective piece, Contribution 16 described in detail the strategy that led to advancing the killed whole-cell ETEC vaccine ETVAX, with and without the mucosal adjuvant dmLT, and transforming it into an ongoing field efficacy trial involving West African children (Table 2). This is a phase 2b field efficacy trial of 6–18-month-olds in the Gambia. It serves as a precursor to a pivotal phase 3 trial currently being planned.

## 7. Assessing the Value of Vaccines

The phrase “Value of Vaccines” refers to the process of estimating the value and public health impact of a vaccine, a process critical to ensuring evidence-based policy decisions are made in the pre- and post-licensure stages [8]. The World Health Organization (WHO) has further extended the concept to the Full Value of Vaccine Assessments (FVVA), identifying the key elements of (1) assessment, (2) decision-making, and (3) communication, all in a framework that provides guidance for stakeholders organizing global efforts to promote investment in vaccines that are priorities for LMICs [9,10].

The exciting technical developments described in this Special Issue are the result of the involvement and collaboration of scientists, donors, and manufacturers. However, for these efforts to ultimately be successful, the active participation of the WHO and other stakeholders is necessary. An important strategic goal of the WHO is to facilitate the development of an effective, safe, inexpensive ETEC vaccine that reduces morbidity and mortality caused by moderate-to-severe diarrhea for administration to infants and children under the age of 5 in LMICs.

By defining the rationale and mapping the key steps needed to facilitate ETEC vaccine development and testing, the WHO and other public health stakeholders are streamlining and facilitating the process of ETEC vaccine licensure.

Without this leadership provided by the WHO and its ability to include other public health stakeholders, the positive progress made in better defining the ETEC disease threat and designing and evaluating promising vaccine candidates would likely have been impossible. WHO guidance documents and publications serve as a bridge between technical development and delivery to target populations and inform the strategies of funders and other public health stake holders.

The ETEC vaccine landscape was comprehensively reviewed in 2006 by a WHO review panel at a time when data from vaccine trials in Bangladesh, Egypt, and Israel had become available. Improving the ETEC vaccines’ immunogenicity and efficacy were the key recommendations of the committee [11]. In 2018, a fresh review of the ETEC field was conducted in recognition of the high levels of both ETEC disease and economic burden, serving as part of a WHO Product Development for Vaccine Advisory Committee (PDVAC) meeting [1,2,12]. As a result of this meeting, ETEC was re-affirmed as a vaccine target priority pathogen. The WHO formulated a working group of experts in this field desiring to better quantify the ETEC vaccines’ public health value and propose improvements to generate improved global burden datasets and methodologies, with a series of analyses and articles ensuing [1,2,12].

Among their recommendations, this expert working group identified ETEC along with *Campylobacter jejuni*, Norovirus, and *Shigella* (*dysenteriae* and *flexneri*) as four priority organisms to target in further research to determine their impact on growth and cognitive development in infants and young children, as well as their roles in contributing to both short- and long-term negative health outcomes in adults from LMICs [1,2,11,13,14].

Guidance specifically for ETEC vaccine developers has been provided by the WHO through Preferred Product Characteristics publications. Within the WHO’s AMR Action Framework, the potential value of an ETEC vaccine for combatting AMR has been assessed [13,15]. Importantly, additional publications have been generated to cover research needs and gaps for ETEC vaccines [1,2,13,14].

## 8. Conclusions

While significant progress has been made in ETEC vaccine development, the need for sufficient and sustained funding remains the most critical barrier to rapidly advancing the worthiest vaccine candidates toward in-country licensure and subsequent pre-qualification through the WHO [1,2,12].

The long lead times of vaccine development impact the timelines of ETEC vaccine advancement, including with respect to the high costs of clinical testing. Changing donor priorities also make it very difficult to move efficiently from one stage of development to the next. Multiple donors will likely be required to push an enteric product forward in a coordinated fashion, something that is difficult to accomplish. In addition, the early involvement of a manufacturing partner can accelerate the success of the overall vaccine program, which is often a challenge due to risks and the perception of the market for enteric vaccines.

As the articles included in this Special Issue on ETEC show, significant progress is being made towards making safe, effective, and practical ETEC vaccines possible. We must consider that greater levels of commitment and coordination not presently apparent may be required before the promise of acceptable vaccines against ETEC and other enteric pathogens can become a reality. The considerable benefit of the realization of this potential is too great to ignore.

## Figures and Tables

**Table 1:** Contributions to this Special Issue on Enterotoxigenic *E. coli* (ETEC) vaccines.

Contribution #, First Author	Focus of Research	Geographic Area of Study or in Which Study Samples Were Collected
Epidemiology and Global Burden		
1. Héma	A rapid Loop-Mediated Isothermal Amplification (LAMP) diagnostic test (RDLT) was applied to diarrheal and non-diarrheal stool samples.	Burkina Faso
2. Mwape	Stool samples from five peri-urban health facilities were tested for diarrheagenic *E. coli*.	Lusaka, Zambia
3. Chakraborty	In this hospital-based study, patients presenting with acute diarrhea were tested for ETEC at two geographically distinct sites.	Rural Bangladesh
4. Jojura	ETEC samples derived from diarrheal and environmental water samples were analyzed for their antimicrobial susceptibility patterns.	Rural Bangladesh
Host Parameters and Genomics that Predict Responses		
5. Kuhlmann	Serum samples were screened for IgG and IgA responses to ETEC LT and the non-canonical proteins EatA and EtpA.	Egypt, Cameroon, Peru, Haiti
6. Girardi	Serum was collected from ETEC-infected adults and children and tested for anti-toxin responses, including neutralization activity, and via linear epitope mapping.	Bangladesh
7. Hollifield	Phagocytosis of ETEC by macrophages and the impact of LT and ST on this process, including with respect to the survival of phagocytosed ETEC, were investigated.	US
8. Bronstad	Plasma levels of the prohormones of guanylin and uroguanylin (which ST mimics) were measured in subjects participating in an ETEC CHIM study.	Norway
Application of New Omics Technologies for the Characterization of Host Responses		
9. Mwape	A new pan-diarrheagenic *E. coli* proteomic array was tested with serum samples from the Zambian phase 1 ETVAX clinical trial.	Lusaka, Zambia
Preclinical Evaluation of Vaccine Candidates and Models of Disease		
10. Li	Polyvalent adhesin-toxoid multiepitope-fusion-antigen (MEFA IIb)-containing epitopes of seven ETEC antigens were tested in mice and rabbits.	US
11. Westcott	Development of a novel ETEC vaccine inactivation method that was evaluated in mice.	US
Vaccine Candidates in Clinical Trials and in Human Challenge Models		
12. Maier	Validated a recently developed disease severity score with alternative clinical endpoints to assess the efficacy of the earlier formulation of ETVAX for travelers.	Guatemala, Mexico
13. Talaat	Vaccination of subjects with the Valneva ETEC/Shigella vaccine followed by ETEC CHIM.	US
14. Guitierrez	A phase 1 clinical trial with ETEC CFA/I fimbrial adhesin antigen with and without mLTmucosal adjuvant.	US
15. Guitierrez	Tested the above vaccine in individuals subjected to an ETEC challenge to determine efficacy.	US
16. Hossain	Description of the strategy for the ongoing phase 2b field efficacy trial of ETVAX, with and without dmLT.	The Gambia

**Table 2:** Current ETEC vaccine candidates, both clinical and preclinical (for further reading, see [1,2]).

Candidate (Developer)	Description	Antigenic Composition	Clinical Status	Reference *
ETVAX (Scandinavian BioPharma)	Oral, inactivated ETEC whole-cell	CFA/I, CS3, CS5, CS6, LCTBA (hybrid of CTB and LT), dmLT	Phase 3	C 1
Fimbrial tip adhesins (Naval Medical Research Institute)	Intramuscular, subunit	CfaEB/CssBA/mLT/dmLT	Phase 2b	C 14, 15 [3]
CVD 12208S-122 (Center Vaccine Development and Global Health, University Maryland)	Oral attenuated *Shigella flexneri* 2a expressing ETEC colonization factors	*S. flexneri* 2a expressing CFA/I and ETEC LT	Phase 1	[4,5]
ShigETEC (Eveliqure Biotechnologies)	Oral attenuated Shigella without OPS or T3SS but expresses ETEC ST and LT toxoids	Natural surface proteins of Shigella as well as ETEC ST and LT	Phase 1	[6]
VLA1701 (Valneva)	Oral inactivated *V. cholerae* with recombinant cholera toxin B subunit (rCTB)	*V. cholerae* 01 Inaba, El Tor, *V. cholerae* 01 Ogawa, classical, rCTB	Phase 1	C 13
MecVax MEFA (University Illinois Urbana-Champaign)	Intramuscular, subunit proteins	CS1-CS7, CS12, CS14, CS17, CS21, LT, ST	Preclinical	C 10 [7]
Non-canonical ETEC proteins (Department Medicine, Washington University)	Intramuscular, subunit proteins	EtpA/EatA	Preclinical	C 9

* Either Special Issue contribution number (C = Contribution) or reference number.

## References

[1] Khalil I., Walker R., Porter C.K., Muhib F., Chilengi R., Cravioto A., Guerrant R., Svennerholm A.-M., Qadri F., Baqar S. (2021). Enterotoxigenic *Escherichia coli* (ETEC) vaccines: Priority activities to enable product development, licensure, and global access. Vaccine.

[2] Khalil I., Anderson J.D., Bagamian K.H., Baqar S., Giersing B., Hausdorff W.P., Marshall C., Porter C.K., Walker R.I., Bourgeois A.L. (2023). Vaccine value profile for enterotoxigenic *Escherichia coli* (ETEC). Vaccine.

[3] Lee T., Gutierrez R.L., Marciel M., Poole S., Testa K.J., Trop S., Duplessis C., Lane A., Riddle M.S., Hamer M. (2001). Safety and immunogenicity of intramuscularly administered CS6 subunit vaccine with a modified heal-labile enterotoxin from enterotoxigenic *Escherichia coli*. Vaccine.

[4] Shigella CVD 31000: Study of Responses with Shigella-ETEC Vaccine Strain CVD 12208S-122. NCTC04634513. https://classic.clinicaltrials.gov/ct2/show/NCT04634513.

[5] Medeiros P.H., Bolick D.T., Ledwaba S.E., Kolling G.L., Costa D.V.S., Oria R.B., Lima A.A.M., Barry E.M., Guerrant R.L. (2020). A bivalent vaccine confers immunogenicity and protection against *Shigella flexneri* and enterotoxigenic *Escherichia coli* infections in mice. NPJ Vaccines.

[6] Harutyunyan S., Neuhauser I., Mayer A., Aichinger M., Szijarto V., Nagy G., Nagy E., Girardi P., Malinoski F.J., Henics T. (2020). Characterization of ShigETEC, a novel live attenuated combined vaccine against Shigellae and ETEC. Vaccines.

[7] Jones R.M., Seo H., Zhang W., Sack D.A. (2022). A multi-epitope fusion antigen candidate vaccine for enterotoxigenic *Escherichia coli* is protective against strain B7A colonization in a rabbit model. PLoS Negl. Trop. Dis..

[8] WHO Immunization Analysis and Insights, Value of Vaccines. https://www.who.int/teams/immunization-vaccines-and-biologicals/immunization-analysis-and-insights/vaccine-impact-value.

[9] WHO Immunization Analysis and Insights, Full Value of Vaccines Assessment. https://www.who.int/teams/immunization-vaccines-and-biologicals/immunization-analysis-and-insights/vaccine-impact-value/value-of-vaccines#:~:text=The%20Value%20of%20Vaccines%20team%20together%20with,Advisory%20Committee%20provides%20advice%20on%20methods%20and.

[10] Hutubessy R., Lauer J.A., Giersing B., Sim S.Y., Jit M., Kaslow D., Botwright S. (2023). The Full Value of Vaccine Assessments (FVVA): A framework for assessing and communicating the value of vaccines for investment and introduction decision-making. BMC Med..

[11] World Health Organization (2006). Future directions for research on enterotoxigenic *Escherichia coli* vaccines for developing countries. Wkly. Epidemiol. Rec..

[12] WHO’s 5th Product Development for Vaccines Advisory Committee (PDVAC) Meeting 26–28 June 2018, Executive Summary. https://www.who.int/publications/m/item/pdvac-executive-summary-2018-5th-annual-meeting.

[13] WHO Preferred Product Characteristics for Vaccines against Enterotoxigenic *Escherichia coli*. https://iris.who.int/bitstream/handle/10665/341507/9789240021839-eng.pdf?sequence=1.

[14] Hasso-Agopsowicz M., Lopman B.A., Lanata C.F., Rogawski-McQuade E., Kang G., Prudden H.J., Khalil I., Platts-Mills J.A., Kotloff K., Jit M. (2021). World Health Organization Expert Working Group: Recommendations for assessing morbidity associated with enteric pathogens. Vaccine.

[15] WHO PPC: For Enterotoxigenic *Escherichia coli* (ETEC) Vaccines. https://www.who.int/tools/target-product-profile-database/item/ppc--for-enterotoxigenic-escherichia-coli-(etec)-vaccines.

